# Pretreatment of Vine Shoot Biomass by Choline Chloride-Based Deep Eutectic Solvents to Promote Biomass Fractionation and Enhance Sugar Production

**DOI:** 10.3390/bioengineering11090935

**Published:** 2024-09-18

**Authors:** Raquel Cañadas, Aleta Duque, Alberto Bahíllo, Raquel Iglesias, Paloma Manzanares

**Affiliations:** 1 Advanced Biofuels and Bioproducts Unit, Department of Energy, Research Centre for Energy, Environment and Technology (CIEMAT), 28040 Madrid, Spain; raquel.canadas@ciemat.es (R.C.); p.manzanares@ciemat.es (P.M.); 2 Sustainable Thermochemical Valorization Unit, Department of Energy, Research Centre for Energy, Environment and Technology (CIEMAT), 28040 Madrid, Spain

**Keywords:** agricultural residue, biomass fractionation, green solvent, enzymatic hydrolysis, lignin

## Abstract

Vine shoots hold promise as a biomass source for fermentable sugars with efficient fractionation and conversion processes. The study explores vine shoots as a biomass source for fermentable sugars through pretreatment with two deep eutectic solvents mixtures: choline chloride:lactic acid 1:5 (ChCl:LA) and choline chloride:ethylene glycol 1:2 (ChCl:EG). Pretreatment conditions, such as temperature/time, solid/liquid ratio, and biomass particle size, were studied. Chemical composition, recovery yields, delignification extent, and carbohydrate conversion were evaluated, including the influence of washing solvents. Temperature and particle size notably affected hemicellulose and lignin dissolution, especially with ChCl:LA. Pretreatment yielded enriched cellulose substrates, with high carbohydrate conversion rates up to 75.2% for cellulose and 99.9% for xylan with ChCl:LA, and 54.6% for cellulose and 60.2% for xylan with ChCl:EG. A 50% acetone/water mixture increased the delignification ratios to 31.5%. The results underscore the potential of this pretreatment for vine shoot fractionation, particularly at 30% solid load, while acknowledging the need for further process enhancement.

## 1. Introduction

The implementation of a real bioeconomy is essential to tackle the global challenges that humanity faces for the next decades. Bioeconomy focuses on an integral use of sustainable biomass resources to provide a great variety of fuels and bio-based products. The biomass conversion process would be carried out in a biorefinery-type facility for the combined production of energy carriers and other bio-based products with applications in the food, feed, pharmaceuticals, chemicals, and materials industries [1]. Among the different biorefinery schemes previously defined [2], the biorefinery system that contemplates the use of lignocellulosic feedstocks, the so-called lignocellulose-based biorefinery, has been intensively investigated in recent decades, resulting in a significant progression in this field [3].

Lignocellulosic biomass (LCB) covers a broad range of materials from different origins (i.e., forest and agro-residues, energy crops, industrial wastes, and municipal solid wastes) including agricultural residues, which constitute a wide source of renewable feedstocks susceptible to revalorization through adequate transformation processes. Vine shoot (VS), the residue originating from the pruning operations of grape crops, represents an abundant and cheap source of residual lignocellulosic biomass. Nonetheless, it currently has limited use as a fuel in power generation industries and mainly in domestic applications [4,5]. The vineyard is a crop of considerable economic importance that yearly produces a great quantity of agricultural residues, particularly VS. According to the Food and Agriculture Organization Statistics (FAOSTAT), close to 6.8 million hectares were cultivated in the world in 2022, with approximately 51% of the total area being located in Europe. The amount of VS biomass produced varies depending on geographical regions, varieties, planting type, etc. However, the figure of 1.2 tons of these residues per hectare of cultivation could be considered a representative average value [6]. Thus, the revalorization of VS as raw material in a biorefinery can contribute to making a sustainable use of an abundant residue that needs adequate disposal. On the other hand, it can mean a strong drive for the social and economic growth of agricultural areas through the development of novel technological processes for bio-based products that can replace the chemicals obtained from fossil raw materials.

In the bioconversion process of a lignocellulosic material such as VS, the first step to undertake is the break of the natural resisting barrier inherent to biomass using a pretreatment process, which fractionates biomass components and facilitates the accessibility of hydrolytic agents to carbohydrates. A significant number of the pretreatment technologies developed until now are based on the demanding use of conventional solvents that are often regarded as a danger to the environment concerning their synthesis, nature, and disposal [7]. Thus, over the past decade, the interest in the search for alternative, readily available, and environmentally friendly greener solvents that can replace the traditional ones has steadily grown. Among the green solvents of current interest, deep eutectic solvents (DESs) have attracted special attention since they possess interesting features such as low volatility, biodegradability, ample liquid range, non-toxicity, and non-flammability. These solvents, also named low-transition-temperature mixtures (LTTMs) by Francisco and co-workers [8], are comparable to Ionic Liquids (ILs) from the point of view of their physicochemical characteristics. However, they are often considered more advantageous, because they can be easily synthesized from readily available biomaterials, present low toxicity and lesser negative environmental impact, and are cheaper than ILs. Generally, DESs are formed by two or more organic compounds that, in a mixture, have a final melting point much lower than the individual components. One of the components acts as a hydrogen bond donor (HBD) and the other one is the hydrogen bond acceptor (HBA). The hydrogen bond interaction between these two constituents is known to lie behind the formation of the eutectic mixture [7].

Lately, DESs have emerged as an attractive option for the pretreatment of LCB in order to extract lignin and enhance cellulose saccharification [9,10]. DES pretreatment could cleave the hydrogen and ether bonds within lignin/carbohydrate complexes, making it easier to selectively extract lignin [10,11]. Typically, HBAs are quaternary ammonium salts, such as choline chloride (ChCl), and HBDs are compounds such as glycerol, ethylene glycol, urea, carboxylic acids, amides, etc. Amongst the multiple possible blends of HBAs and HBDs, ChCl, derived from biomass, is widely used as an HBA component due to its availability, low cost, biodegradable and non-toxic characteristics, and excellent pretreatment efficiency [12,13,14]. Chloride ions can form hydrogen bonds with hydroxyl groups in polysaccharides and lignin, disrupting the initial intermolecular hydrogen bonds in LCB, which favors pretreatment [13,15]. For HBD, acid-based components and polyols exhibit favorable attributes for lignin extraction by disrupting the bonds within lignin/carbohydrate complexes [11,16,17]. Promising DES for LCB pretreatment include choline chloride/lactic acid (ChCl:LA) and choline chloride/ethylene glycol (ChCl:EG), which have shown positive effects on delignification [9,10,16,17,18,19,20].

However, in spite of the increasing interest in DES use for biomass pretreatment and the abundant literature in this regard published in recent years, the general consensus about DES is that they are still in a nascent stage and further research is needed to fully characterize them and understand their performance [8]. More specifically, the employment of DES in a novel and particular LCB material makes it essential to define the best process conditions leading to an optimum biomass fractionation and a maximum biomass components recovery.

The present work aims to evaluate the effectiveness of ChCl-based DES to fractionate VS biomass, facilitate major biomass components recovery, and generate a suitable pretreated material to be used as a substrate for fermentable sugar production. Special attention is paid to the capability of ChCl-based DES (ChCl:LA 1:5 and ChCl:EG 1:2) to dissolve lignin, recover this component, and maximize sugar release from the carbohydrates contained in pretreated VS through enzymatic hydrolysis. To this end, the selected DES mixtures are tested under different assay conditions of temperature/time, solid/liquid ratio, and biomass particle size. In addition to these parameters, this work encompasses the study of a particular aspect of the pretreatment methodology regarding the influence of the washing solvent (water or 50% acetone/water) in the removal and recovery of lignin after the incubation of the DES/biomass mixtures. The results are evaluated in terms of lignin solubilization and recovery, and the conversion efficiency of the main carbohydrates (cellulose and hemicellulose) in DES-pretreated materials into monomeric sugars through enzymatic hydrolysis. In addition, the lignin-rich material isolated by the DES pretreatment is analyzed through thermogravimetric techniques (TGA) in order to find out the thermal properties of these materials and compare them with a commercial lignin. The pretreatment of vine shoot biomass with the DES mixtures used in the present study has not been previously reported in the literature. In addition, the experimental plan introduces the variable particle size and solid loading, usually not considered in this type of study, expecting to enlighten about the possibilities for process intensification.

## 2. Materials and Methods

### 2.1. Biomass

Vine shoots (VSs) were provided by the biomass valorization company VanMander S.L. (located in Santa Margarita, Barcelona, Spain). The original biomass was milled by the Centre for the Development of Renewable Energy Sources (CEDER) (Soria, Spain) to a final particle size of 2 mm and a moisture content of 8.0 ± 0.0%. The milled VS were then sent to CIEMAT laboratories in Madrid for the experimental work. A biomass sample was further crushed to about 1 mm particle size (moisture content of 6.8 ± 0.0%) using a laboratory sample mill (Cyclotec 1093, Foss A/S, Hilleroed, Denmark) for composition analysis. Likewise, another part was milled again to 0.5 mm particle size for DES pretreatment experiments on the effect of particle size on biomass fractionation and sugar release. All the VS biomass samples were homogenized and stored in an oven at 40 °C until used.

### 2.2. Materials and Chemicals

All the chemicals used in this study were purchased from Sigma Aldrich (Madrid, Spain) and used without further purification. In the case of DES formation, the following compounds were purchased: choline chloride (ChCl, purity ≥ 98% weight/weight (*w*/*w*)), lactic acid (LA, purity ≥ 85% *w*/*w*), and ethylene glycol (EG, purity ≥ 99.8% *w*/*w*). Then, the DESs were prepared according to the procedure reported in Section 2.3 below.

For the enzymatic hydrolysis assays, a commercial enzyme cocktail (SAE0020, Sigma-Aldrich Co, Spain, enzyme activity 150 FPU/mL), containing a mixture of cellulases, ß-glucosidases, and hemicellulases, was used. In addition, sodium citrate (purity ≥ 99.5% *w*/*w*), sodium azide (purity ≥ 99.5% *w*/*w*), and Tween^®^ 20, all of them purchased form Sigma Aldrich (Spain) were added to the enzyme media. The commercial organosolv lignin, used for comparative purposes in TGA analysis, was also obtained from Sigma Aldrich (Spain).

### 2.3. DESs Preparation

Two deep eutectic solvents with different characteristics were chosen and prepared with the aim to evaluate them as the pretreatment agents of VS. Choline chloride (ChCl) was chosen as the hydrogen bond acceptor (HBA) and it was combined with one of two renewable hydrogen bond donors (HBD), either a monocarboxylic acid (lactic acid (LA)), or a polyalcohol (ethylene glycol (EG)). The resulting mixtures are referenced as ChCl:LA and ChCl:EG, respectively. Firstly, each component was precisely weighed according to the corresponding molar ratio of 1:5 for ChCl:LA and 1:2 for ChCl:EG to be placed and mixed in a 100 mL glass beaker. The mixed components were kept at 60–80 °C with a stirring rate of 350 rpm for 60–120 min until uniform and transparent liquid was formed. The selected molar ratios and preparation conditions were carried out according to the literature [21,22,23]. Finally, homogeneous mixtures were obtained in the form of DESs, which were kept in an oven at 40 °C until use.

### 2.4. Solvent Pretreatment

The VS biomass and the corresponding DES were added to a 50 mL round-bottomed pressure glass tube and completely mixed with the help of mechanical stirring. Then, the effect of the three influential variables solid loading, biomass size, and incubation temperature/time combinations were studied. The mixing was carried out in different proportions, increasing the solid loading to be evaluated in each assay (from 5% to 10% and finally, to 30%). The loading was not increased further because otherwise a good transfer and complete mixing of the biomass sample with the DES could not be achieved. Also, in a novel way that has not been explored in other similar studies, two biomass particle sizes, 0.5 mm and 2 mm, were evaluated. After blending the mixture under study in each assay, the tubes were placed in a thermostatic bath (Bath circulator BN3, Thermo Haake, Karlsruhe, Germany) at different temperature/time combinations based on the scientific literature [10,24]. Temperature is a crucial factor for the pretreatment [22] and preliminary studies conducted by the group (not shown) confirmed that it was necessary to work above 110 °C to effectively alter the biomass. Thus, for ChCl:LA, tests were performed at 120 °C for 6 h, or at 150 °C for 3 h, while in the case of ChCl:EG, the incubation time was 17 h for both temperatures.

Table 1 summarizes the conditions evaluated in the DES-based pretreatment assays performed for temperature, solid loading, and particle size. In the ChCl:EG batch of experiments, the temperature of 120 °C with a 30% solid loading was not tested, as previous assays carried out in this study had shown unsatisfactory pretreatment performance under these particular conditions.

**Figure 1:**
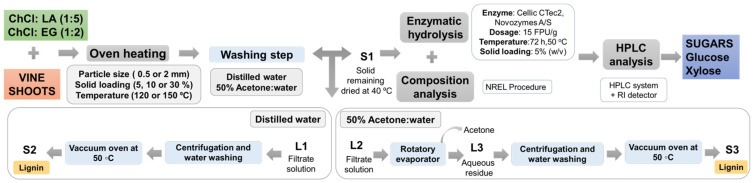
Schematic diagram of solvent pretreatment in this study. Abbreviations and nomenclature definition: ChCl: LA, choline chloride/lactic acid; ChCl:EG, choline chloride/ethylene glycol; S1, solid sample after pretreatment and washing step; L1, filtrate solution with distilled water; L2, filtrate solution with 50% acetone/water; L3, aqueous residue; S2, lignin-enriched solid after water washing; S3, lignin-enriched solid after 50% acetone/water washing.

After the time for each assay had elapsed, the tubes were removed from the bath and, after cooling at room temperature, the mixtures were washed with distilled water and filtered under vacuum until the filtrate solution had a pH and conductivity close to that of the distilled water. The pH was measured with pH indicator strips and a conductivity test was carried out using a conductivity meter (HI5522 multi-parameter pH/ORP/ISE/EC meter, HANNA Instruments, Padova, Italy). A diagram of the separation and washing process is depicted in Figure 1. The solid samples remaining in the filter (hereinafter, S1) were weighed and oven-dried at 40 °C for 24 h (for ease of handling and to avoid possible contamination) to submit them to composition analysis and preserve them until the subsequent enzymatic hydrolysis was carried out, as described in detail in Section 2.5 and Section 2.6 below, respectively.

The filtrate solution obtained (L1) was centrifuged to obtain a second solid residue using a Universal 320R centrifuge (Hettich, Kirchlengern, Germany). This residue was washed twice with water and dried in a vacuum oven (Heraeus VT 5042 Vacuum Oven, Spain), obtaining the solid called S2 (lignin-enriched solid). The weight of S2, together with the weight of S1, was used to estimate the solid recovery (Equation (1)).

(1) $$Solid\;recovery\left(\%\right)=\frac{\left(m_{1}+m_{2}\right)}{m_{0}}\times 100$$

where m_0_ is the initial mass (g) of the solid sample, m_1_ is the final mass (g) of the solid sample after the pretreatment and washing step (S1), and m_2_ is the solid recovered after the centrifugation step (S2).

In addition, the percentage of delignification was estimated (Equation (2)), which was calculated as a function of the percentage of lignin present in the recovered sample mass compared to the amount present in the initial sample.

(2) $$Delignification\left(\%\right)=100-\left[\frac{\left(m_{1}\times C_{lig,m1}\right)}{\left(m_{0}\times C_{lig,m0}\right)}\times 100\right]$$

where m_0_ is the initial mass (g) of the solid sample, m_1_ is the final mass (g) of the solid sample after pretreatment and washing step (S1), and C_lig,m0_ (%), and C_lig,m1_ (%) are the lignin concentration in each sample.

#### Testing of an Alternative Washing Methodology

An alternative solvent for the washing of the pretreated material was also tested in the experiments at 30% solid loading and 0.5 mm particle size. These experiments were aimed at improving the results of lignin removal by promoting a more thorough wash of the DES pretreated solid since the first tests in the selected conditions had shown poor performance. The selected washing solvent was a solution of acetone/water (50% v/v), based on the idea that acetone is frequently reported as an efficient washing agent in the literature [25,26,27]. In this case, the acetone was evaporated from the filtered solution in a rotary evaporator (Hei-VAP Core HL G3 XL, Heidolph Instruments GmbH & Co. KG, Schwabach, Germany) in order to precipitate the recovered lignin and then the so obtained aqueous residue (L3) was subjected to centrifugation to finally recover the lignin in solid form (S3). The methodology applied is also described schematically in Figure 1.

### 2.5. Compositional Analysis of Samples

The untreated VS biomass and every DES-pretreated sample were analyzed according to the methodology followed by the National Renewable Energy Laboratory (NREL, Golden, CO, USA) for biomass analysis as described by Sluiter et al. [28]. The analysis involves quantifying the main components of the biomass, including cellulose, hemicellulose, acid-insoluble lignin, acid-soluble lignin, and acetyl groups. For that purpose, the samples undergo two acid hydrolysis steps, first with 4% H_2_SO_4_ at 121 °C for 30 min and then in an autoclave with diluted acid at 121 °C for 60 min. After cooling and filtration, the liquid was analyzed for sugars and acetic acid using High-Performance Liquid Chromatography (HPLC). The particular conditions of the HPLC analysis are described in the work by Moreno and co-workers [29]. Furthermore, acid-soluble lignin was determined by a UV-spectrophotometric analysis, and acid-insoluble lignin in the non-hydrolyzed biomass was determined by weight according to the specific protocols [28].

### 2.6. Enzymatic Hydrolysis

The DES-pretreated and washed solid residue, S1, was used as a substrate for enzymatic hydrolysis (EH) tests. The saccharification was performed in triplicate in 50 mL Erlenmeyer flasks. Samples weighing 0.5 g were added to a 0.05 M sodium citrate buffer solution (pH 4.5), resulting in a consistency of 5% (*w*/*w*) of solids. The hydrolysis medium also contained an enzymatic cocktail dose of 15 FPU/g substrate, 10% (v/v) of sodium azide (NaN_3_) to prevent contamination, and 0.125% (v/v) of surfactant Tween^®^ 20 to favor the substrate/enzyme interaction. The flasks were incubated in an orbital shaker incubator (Minitron, Infors HT, Bottmingen, Switzerland) at 50 °C and 150 rpm for 72 h. Following the incubation period, an aliquot was withdrawn from each flask. The aliquots were then centrifuged at 13,000× *g* for 10 min, and the supernatant was diluted five times for the analysis of sugar content. HPLC was used to quantify the concentration of soluble sugars in the samples. The HPLC method used a Waters 2695 chromatograph with a CHO-682 LEAD column at 75 °C and Milli-Q water at 0.5 mL/min as the mobile phase [29]. Specifically, the sugars analyzed included glucose, xylose, galactose, arabinose, and mannose.

The efficiency of EH was evaluated by calculating cellulose and xylan conversion yields (CY (%) and XY (%), respectively), according to Equations (3) and (4) below. The yields are based on the sugars released in the EH media by the enzyme action and thus, the quantity of glucose measured in the hydrolysis medium is corrected with the amount of glucose contained in the enzymatic cocktail.

CY (%) = [(C_glu,EH_ − C_glu,enz_) × V_EH_]/[m × (C_gu,substrate_/100)] × 100 (3)

XY (%) = (C_xyl,EH_ × V_EH_)/[m × (C_xyl,substrate_/100)] × 100 (4)

where C_glu,EH_ (g/L) and C_xyl,EH_ (g/L) are the final concentration of glucose and xylose in hydrolysis medium at 72 h; C_glu,enz_ (g/L) is the glucose concentration in the enzymatic cocktail; V_EH_ is the volume of the enzymatic media; m (g) is the dry DES-pretreated sample subjected to hydrolysis; and C_glu,substrate_ (%) and C_xyl,substrate_ (%) are the equivalent glucose and xylose content in the DES-pretreated sample, respectively.

### 2.7. Thermogravimetric Analysis

The thermogravimetric analysis (TGA) was performed using Mettler TGA2 equipment (Mettler-Toledo S.A.E., Barcelona, Spain), which measures and records mass and temperature changes over time. The samples employed for TGA experiments were previously ground. About 10–15 mg sample was placed on a crucible of alumina oxide of 70 mL avoiding contact with both sides of the oven. Prior to TGA, temperature, mass, and platform calibrations were carried out. The total N_2_ flow was set to 50 mL/min, with a heating rate of 20 °C/min, and the temperature was heated from room temperature to 900 °C for all the samples. N_2_ was used as TGA purge gas with a flow rate of 20 mL/min. The set of experiments was conducted using N_2_ to analyze the effect of pyrolysis on the composition of lignin.

## 3. Results and Discussion

### 3.1. Chemical Composition of VS Biomass

The chemical composition of the VS biomass was determined as explained in Section 2.5 above, and the results are listed in Table 2 below. As shown, VS is a lignocellulosic biomass that contains approximately 50% (dry weight basis, dwb) of carbohydrates, of which 32.3% are cellulose and 18.4% hemicelluloses. Hemicelluloses are mostly made up of xylan, accounting for close to 15% of the raw VS, and minor quantities of other polymers, such as galactan, mannan, and arabinan, with values ranging from 1.9 to 0.7% (dwb). A significant acetyl group content of 5.3% is also found, which indicates a highly acetylated xylan structure. In relation to the lignin content, the value of 26.6% corresponds to a relatively high lignified biomass. The other minor components quantified are ash (1.6%) and roughly 9% of extractives (close to 7% water, and 1.4% ethanol extractives, with a glucose content of 0.3%). As a whole, VS can be considered a material with a high potential to be used as feedstock for bio-based compound production considering its relatively high carbohydrate content and the presence of other valuable components such as lignin and extractable material.

### 3.2. Effect of DES Pretreatment Conditions on Chemical Composition of VS Biomass

**Figure 2:**
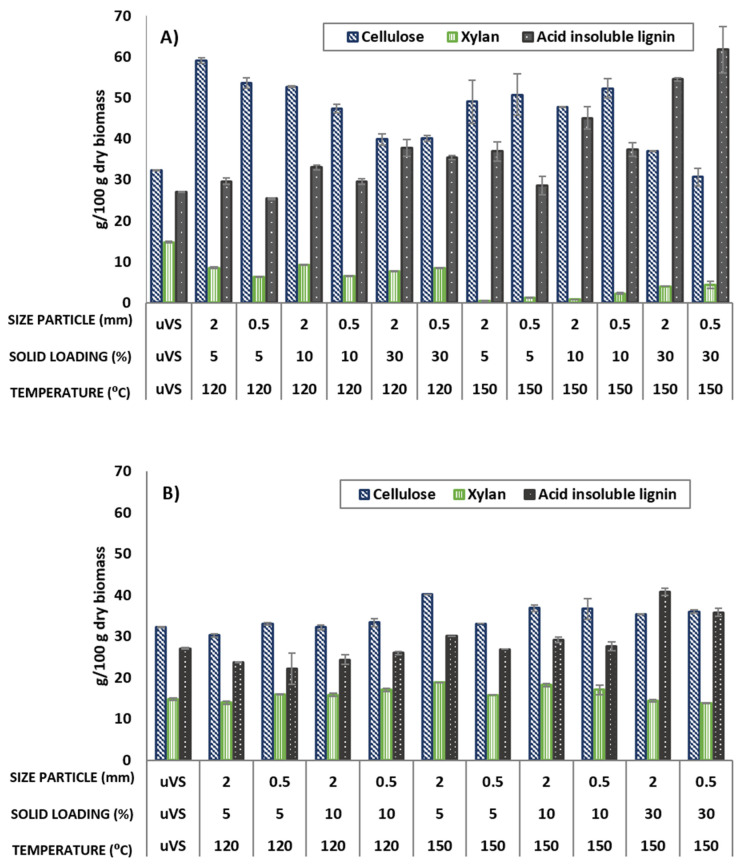
Main components: cellulose, xylan, and acid-insoluble lignin (calculated as g/100 g of dry biomass, (%) of the solid residues pretreated using different DESs: (**A**) ChCl:LA (1:5) and (**B**) ChCl:EG (1:2). uVS = untreated VS biomass.

Firstly, the effect of different test conditions on the chemical composition of the samples after the pretreatment of the VS biomass with the eutectic solvents under study was evaluated. The operating conditions studied have a significant weight on the efficiency of the pretreatment through the changes induced in the structure and chemical composition of the material. The results of the composition in the main components of the S1 solids are depicted in Figure 2 below, which also includes the untreated VS biomass values for comparison purposes. The three key variables most significantly influenced by the DES pretreatment were cellulose, xylan, and lignin content.

In the case of using ChCl:LA, Figure 2A, it was observed that as the biomass was subjected to more severe conditions, cellulose and acid-insoluble lignin became more concentrated, while the proportion of xylan decreased. This effect is due to the solubilization of a great part of the xylan present in raw VS, as well as other non-structural components such as extractives, soluble ash, etc. In particular, the maximum values of cellulose of up to 59.1% (under the conditions of 2 mm—5% solid load—120 °C), acid-insoluble lignin of up to 54.5% (under the conditions of 0.5 mm—30% solid loading—150 °C), and xylan minimums of 0.5% (under the conditions of 2 mm—5% solid loading—150 °C) were obtained. These values can be compared with the cellulose and xylan values in the untreated samples of 32.3%, 26.0%, and 14.8%, respectively. The xylan content exhibits a decreasing trend with increasing temperature. This phenomenon can plausibly be ascribed to a higher progressive cleavage of the lignin/carbohydrate bonds as the treatment severity escalates [30]. In contrast, it was observed that lignin yields increased with elevated temperature and higher solid loading, most likely attributable to the increased destruction of hydrogen bonds within the cell walls [30]. In addition, the high amount of lignin observed in the latter tests may be due to the formation of pseudo-lignin, which may have caused a positive bias in the compositional analysis of lignin. Decomposed carbohydrates have been reported to form lignin-like structures called pseudo-lignin when the biomass is pretreated at severe conditions (e.g., high temperature, long reaction time, or high acidity), as well as with the use of DES formed by a chloride anion [31,32].

As shown in Figure 2B, the VS biomass pretreatment with ChCl:EG does not seem to have as much influence on the composition of the samples as with ChCl:LA. The amount of cellulose, xylan, and acid-insoluble lignin in S1 was observed to be quite stable during the proposed DES pretreatment, mostly in experiments at 120 ºC. However, at a higher temperature of 150 °C, significant variations are found, with the cellulose values increasing up to 40.2 ± 0.8% (at 2 mm—5% solid loading—150 °C conditions), acid-insoluble lignin reaching up to 40.8 ± 4.5% (at 2 mm—30% solid loading—150 °C conditions), and xylan decreasing to 13.8 ± 0.1% (at 0.5 mm—30% solid loading—150 °C conditions). As discussed above for the ChCl:LA experiments, this “concentration” effect occurs at the expense of the breakdown and solubilization of a part of xylan-type polymers, as well as non-structural components.

### 3.3. Effect of DES Pretreatment Conditions on Biomass Fractionation

**Figure 3:**
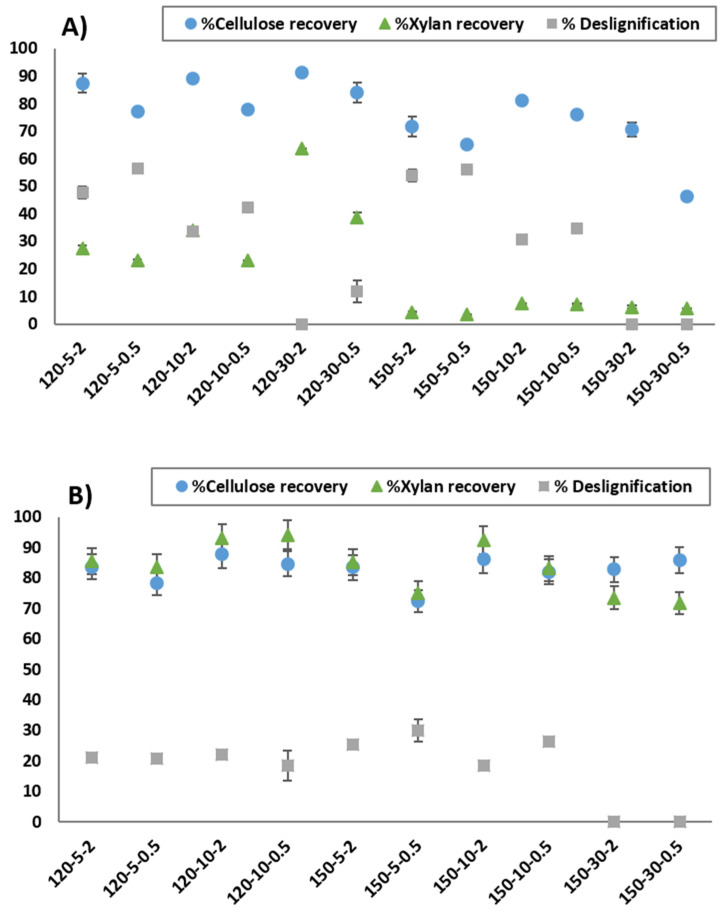
Carbohydrate (cellulose and xylan) recovery (%) and lignin removal rate (%) of pretreated samples using different DESs: (**A**) ChCl:LA and (**B**) ChCl:EG.

The influence of the assay conditions on the recovery of solids and the delignification of the raw VS is shown in Figure 3. As depicted, the pretreatment with ChCl:LA (Figure 3A) yielded more pronounced effects in comparison to ChCl:EG pretreatment (Figure 3B). It is notable that recoveries exhibited a more stable trend during the proposed ChCl:EG pretreatment, in accordance with the results found in the chemical composition of solids.

In a broader context, the degradation of cellulose and xylan is accentuated by rising temperatures, signifying the dissolution of more biomass components at a higher temperature of 150 °C. When considering the outcomes in the context of the effect of particle size, it becomes evident that the use of smaller particles, specifically 0.5 mm, tends to reduce carbohydrate recovery in most cases, with the most significant impact being observed in the case of ChCl:LA.

Regarding delignification, the DES used, and the solid load plays a decisive role in lignin removal. Pretreatment with ChCl:LA demonstrated superior delignification performance. DES with an acid-based HBD usually exhibits better performance in lignin extraction as proton-catalyzed bond cleavage is the principal mechanism in delignification [33]. Moreover, it could be observed that an increase of up to 30% of solid load was adverse for the removal of lignin. The maximum delignification values achieved were up to 56.2% for ChCl:LA and 29.9% for ChCl:EG under the conditions of 0.5 mm biomass size, 5% solid load, and 150 °C, highlighting again the role of the chemical structure of the selected DES.

Furthermore, a chemical compositional analysis of the lignin samples (S2) from which a substantial quantity was successfully retrieved revealed that the proportion of acid-insoluble lignin in the samples was significant, within the range of 57.9 ± 0.8% to 84.6 ± 1.2% (data not shown). It should be taken into account that the values obtained could be influenced by the possible presence of residual traces of the DESs that could remain trapped in the recovered and analyzed samples despite the washing step. However, it is important to emphasize that the main goal of this specific study is not simply to achieve exceptionally pure lignin but to optimize its recovery. These results are comparable to those reported in previous studies. Cardoza et al. (2024) achieved a lignin removal of 43% by a sequential acid/organosolv pretreatment of grapevine shoots at 180 °C [34]. While, Dávila et al. (2017) reported a lignin removal of 67.7% by an alkaline delignification process (2% NaOH, 124 °C, 105 min) on pretreated vine shoots [35]. In addition to the percentage of delignification of the samples by the action of the evaluated pretreatment, the rate of solid recovery with respect to the initial mass was also quantified (Table 3). In general, a higher solid recovery was observed after using ChCl:EG compared to ChCl:LA. This, in turn, coincides with the fact that minor alterations in the composition of the samples pretreated with this solvent were observed.

### 3.4. Sugar Production by Enzymatic Hydrolysis of VS after DES Pretreatment

**Figure 4:**
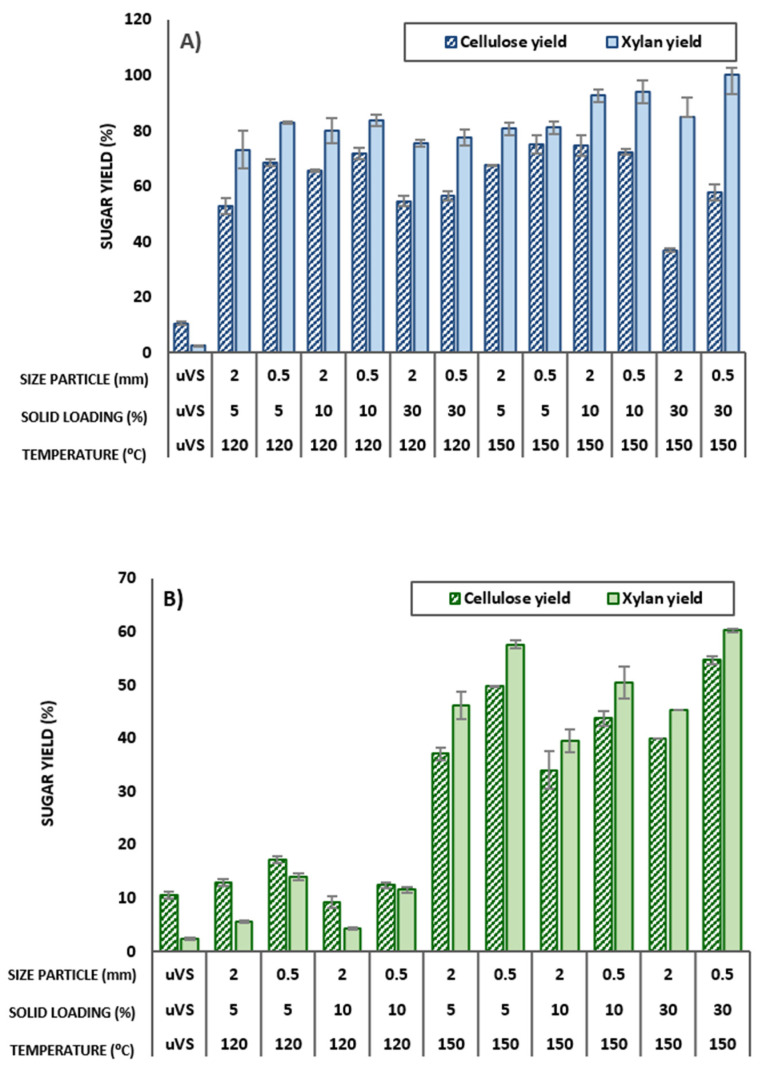
Effect of pretreatment conditions on conversion yield (cellulose and xylan yield (%) calculated by Equation (3) and Equation (4), respectively) after enzymatic hydrolysis of DES-pretreated vine shoots with (**A**) ChCl:LA (1:5) and (**B**) ChCl:EG (1:2). uVS = untreated VS biomass.

The impact of the process conditions of both the ChCl:LA and ChCl:EG pretreatment process on the efficiency of carbohydrate conversion in the pretreated solids by enzymatic hydrolysis is depicted in Figure 4. Focusing on ChCl:LA (Figure 4A), a significant increment of cellulose and xylan conversion yield was found on the whole in comparison with the low value of the untreated VS biomass (10.5 and 2.4% for cellulose and xylan, respectively). The values of cellulose conversion range from 52.7 to 75.2%, the maximum being obtained at 150 °C, 5% solids, and 0.5 mm particle size. Nonetheless, at 120 °C, 10% solids, and 0.5 mm particle size, also reasonably meaningful values close to 72% conversion yield were found. In relation to the xylan conversion yields, high values were found in general, reaching a value of up to 99.9% (at 150 °C, 30% solids, and 0.5 mm particle size). However, the substantially low xylan content of the pretreated substrates must be considered in relation to these results.

Regarding the effect of incubation temperature (T), the conversion of both carbohydrates was positively affected by the increase in T from 120 to 150 °C, with increments in the interval of 10–30% and 9–15% for the cellulose and xylan conversion, respectively, when comparing at the same particle size and solid load. This increment is consistent with the increased solubilization of xylan from the material as T rises (from around 8% at 120 °C to 0.5–4% at 150 °C), which has been demonstrated to positively affect the enzymatic hydrolysis of the cellulose [36]. An exception in the trend in cellulose conversion yield with T occurs when the results at 30% solids are analyzed, since the tendency changes and the increment in T results in no change or even a decrease in the yield, particularly at 2 mm particle size. The decrease in cellulose conversion at 30% solids and 150 °C can be attributed to the elevated lignin content in the solid residues obtained under these process conditions: 54.3% and 61.7%, for 2 and 0.5 mm, respectively, which likely corresponds to pseudo-lignin structures (see Section 3.2). This suggests that in the ChCl:LA experiments, 120 °C is a more optimal pretreatment temperature for the VS pretreatment than 150 °C, which would produce a pretreated material showing lower enzymatic digestibility yields, remarkably at high solid consistencies.

In relation to the xylan conversion in the treatment with ChCl:LA, the effect of T was not so remarkable, and the detrimental effect detected in the cellulose conversion yield at 30% solid and 150 °C was only found in the experiments carried out with 2 mm particle size. About this last case, and in general, it can be stated that the use of a lower particle size, 0.5 mm, in comparison to 2 mm, produces a positive effect on the enzymatic digestibility of the DES-pretreated materials regardless of the temperature tested. This statement is also valid for ChCl:EG, where the use of VS milled to 0.5 mm resulted in average increments of 35% in the cellulose and 180% in the xylan conversion compared to the experiment at 2 mm.

In the case of ChCl:EG (Figure 4B), the maximum conversion values were attained at 150 °C, 5% solids, and 0.5 mm, with close to 50% and 58% conversion for cellulose and xylan, respectively; although unexpectedly, similar results were found at 30% solid conditions. Moreover, using this DES, the positive influence of temperature is very noticeable, with up to 6-fold and 14-fold increments for the cellulose and xylan conversion, respectively. This result is probably related to the fact that increasing the temperature decreases the viscosity of the DES, which tends to improve the pretreatment performance, as previously seen in the literature [33].

The previous calculation of the rate of solid recovery values (Section 3.3) also allows for the determination of the efficiency of enzymatic hydrolysis (EH) with respect to the glucose and xylose content in the untreated VS biomass. For ChCl:LA, the maximum cellulose conversion yields achieved were 69.7% (at 120 °C, 10% solids, and 0.5 mm particle size) and 72.1% (at 150 °C, 5% solids, and 0.5 mm particle size). Focusing on the xylan conversion yields, the maximum value achieved was 44.5% (at 150 °C, 30% solids, and 0.5 mm particle size). These results confirm the different effect of ChCl:LA on the carbohydrate solubilization of the VS. Little cellulose is lost in the pretreatment, whereas an extensive hemicellulose solubilization is observed. In the case of ChCl:EG, the maximum conversion values were 39.3% for cellulose and 43.3% for xylan (both at 150 °C, 5% solids, and 0.5 mm particle size), indicating that this DES has low hemicellulose solubilization potential.

### 3.5. Effect of the Washing Agent

The effect of the washing agent on the sugar production from the DES-pretreated VS biomass was further examined following the washing procedure outlined in Section Testing of an Alternative Washing Methodology above. Acetone solutions, despite having a less favorable environmental profile compared to water, are frequently employed as pretreatment solvents and/or washing agents in the fractionation of lignocellulosic biomass due to their advantageous effects of methyl groups on water/carbonyl association [37]. The acetone/water complex exhibits a pronounced solubility for lignin, and, as an anti-solvent, it has demonstrated the ability to effectively regenerate dissolved lignin [38,39,40]. Aprotic solvents like acetone act as hydrogen acceptors, and the addition of water enhances acetone polarization, thereby facilitating interactions with lignin [41].

Hence, in this study, a 50% acetone/water solution was tested as an alternative to water with the aim of promoting lignin solubilization and the removal of any residues of DES in the pretreated solid. The approach was implemented on two particular pretreated residues (120 °C—30%—0.5 mm with ChCl:LA and 150 °C—30%—0.5 mm with ChCl:EG), in which delignification extent was found to be rather low and thus, susceptible to improvement. The results are shown in Table 4 below.

Regarding the analysis of the results obtained, as anticipated and intended, the use of a solution of acetone as a washing agent significantly enhances delignification for the samples treated with both DES, the recovery and precipitation of lignin showing improvement. Furthermore, there is an increase in solid recovery, possibly due to the enhanced lignin precipitation and the utilization of a reduced washing volume. In terms of the effect on the sample composition, a trend is observed wherein the cellulose proportion is concentrated to a greater extent compared to the use of water. Additionally, there is an increase in the xylan concentration and lignin content compared to the untreated sample. Based on the results, the option of washing with acetone solution appears advantageous as it enhances the delignification yields while maintaining the sugar production values within ranges similar to those obtained after washing with water. Furthermore, both the DES and the washing agents (water and acetone) could be recovered for reuse through the subsequent distillation of the volatile components [39].

### 3.6. Characterization of Recovered Lignin-Rich Solid

The lignin-enriched solids obtained after the ChCl:LA pretreatment of the raw VS biomass carried out in the conditions shown in Table 1, following the experimental procedure summarized in Figure 1 (solid S2), were analyzed by thermogravimetric analysis (TGA) techniques to study the thermal behavior of the samples. For comparison, a commercial organosolv lignin and a sample of the raw VS biomass were also tested.

**Figure 5:**
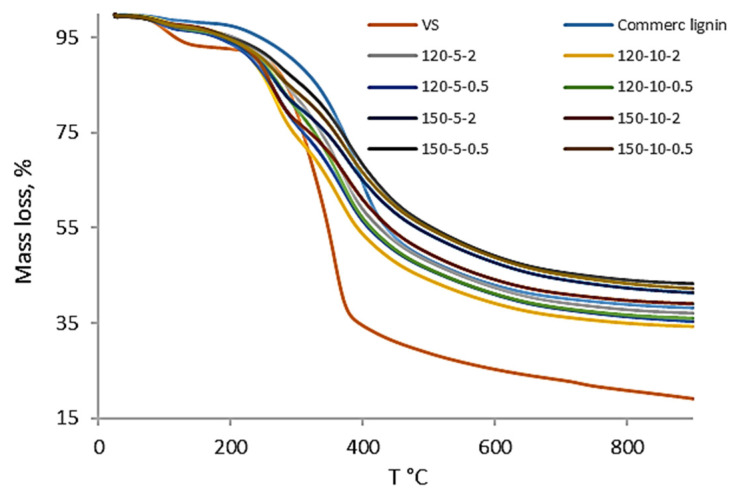
Evolution of the mass loss (g/100 g dry biomass, (%)) of the raw VS biomass, commercial lignin, and the solids S2 at different ChCl:LA (1:5) pretreatment conditions versus temperature (°C).

The changes in the biomass structure after the pretreatment are reflected in the thermal stability studies of the resultant samples. Two representative stability parameters, the mass loss and the decomposition temperature, at which the maximum weight loss per unit of takes place time, were analyzed using the TGA of the pretreated biomass. Figure 5 represents the normalized mass loss (%) of the raw VS biomass, commercial lignin, and the different solids S2 versus temperature (°C).

The mass loss (whose specific values are shown in Table 5 below) for all the S2 solids is between 56% and 65%, very similar to the mass loss of the commercial lignin and far from the 80.8% mass loss of the raw VS biomass. At first glance, it is observed that the samples behave like the commercial lignin (light blue line) and are far from the behavior of the raw VS biomass (orange line). The mass loss of the commercial lignin is similar to that reported in other papers [42,43]. Firstly, there is a small mass loss due to the removal of moisture, then most of the mass loss is between 250 and 450 °C, which corresponds to the thermal decomposition of hemicellulose (260–290 °C) and cellulose (360–380 °C). Lignin could not be distinguished by a specific peak. This was most likely due to the fact that the thermal degradation of lignin occurred throughout the decomposition region of hemicellulose and cellulose (200 to 700 °C) [41] and more specifically with the decomposition of the phenolic and organic compounds of lignin which occurred from 150 to 470 °C [44].

Furthermore, a greater mass loss is observed for the solids S2 at 120 °C (63–66%) than the ones at 150 °C (56–61%). This is influenced by the fact that the degradation of the solid components is accentuated with rising temperatures, signifying the dissolution of more biomass components at a higher temperature of 150 °C. Consequently, there is a higher mass loss experienced by the sample when moving from its initial state to S1, as depicted in Figure 1.

In addition to the solids S2 generated in the pretreatment with ChCl:LA at different reaction conditions discussed above, the sample S3 was also analyzed. This S3 sample was obtained after washing with 50% acetone/water the solid from the test at 120 °C, 30% solids, and 0.5 mm particle size. As pointed out in Section 3.5, in the DES pretreatment experiments at 30% solids the recovery of lignin was rather low and the washing with 50% acetone/water was tested aiming at improving lignin removal. The results of the mass loss and T_max_ of S3 (Table 5) were similar to those in S2 regardless of the washing agent used. Therefore, the solid load and the washing agent are not important factors to take into account in the thermal characteristics of the lignin-enriched samples.

**Figure 6:**
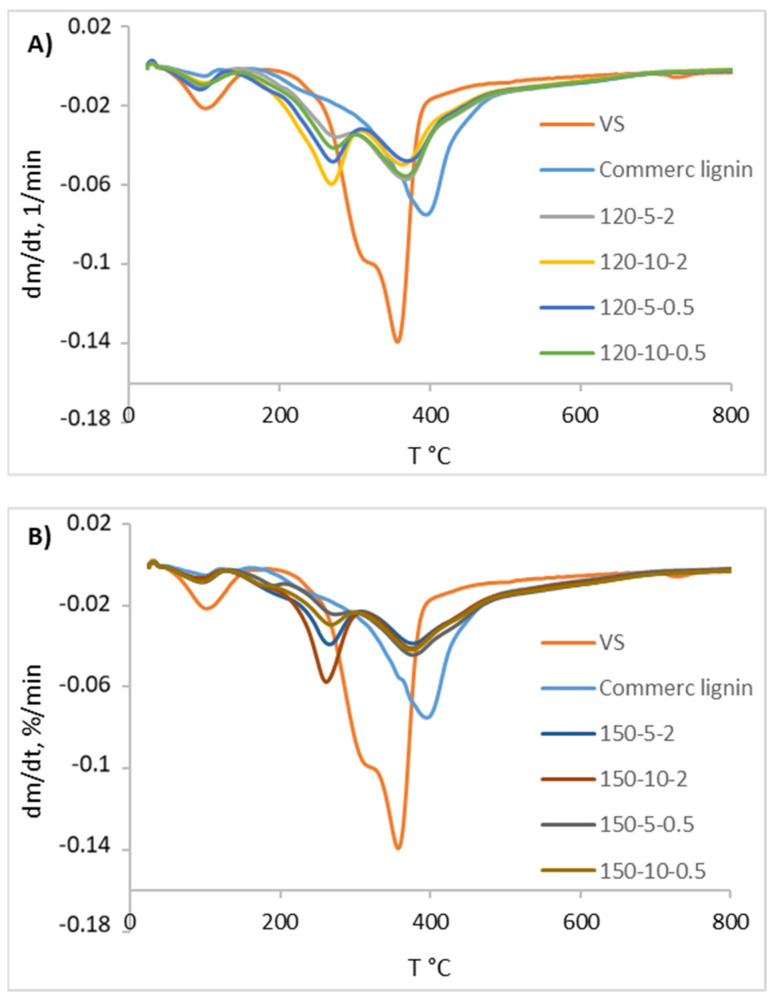
Differential thermogravimetric analysis (DTG) profiles obtained for the samples of ChCl:LA lignin-enriched solids generated at different conditions of solid load and particle size: (**A**) 120 °C; (**B**) 150 °C.

Additionally, the differential thermogravimetric analysis (DTG) profiles (Figure 6) obtained for the solids S2 were used to determine the temperature at which the maximum weight loss (T max) takes place. The figure shows two clearly differentiated zones in the temperature range between 260–280 °C and 365–395 °C. In each zone, the temperature at which the higher mass loss is achieved has been determined and the percentage of mass loss calculated (Table 5).

By analyzing the results presented in Table 5 and Figure 6, the raw VS biomass (orange line) presents the typical behavior of a biomass reaching the highest mass loss at 357 °C. It is clearly observed how the peak of the commercial lignin sample (light blue line) is more displaced to the right (395 °C) and is more pronounced. The rest of the samples (shown in panels A and B of Figure 6) present two clearly differentiated peaks in the temperature range 260–280 °C and in the range 355–395 °C. It can be seen how the samples that present a greater peak in the temperature range 260–280 °C present a lower peak in the temperature range 355–395 °C. Thus, the DES-lignins obtained from the pretreatment at 120 °C are more similar to the commercial lignin used for comparison in this study. Nevertheless, this commercial lignin, coming from an organosolv pretreatment, is more thermally stable than any of the DES-lignins recovered.

Regarding the effect of particle size and solid loading on the pretreated samples, this is not appreciable, or at least in these essays, it has not been clearly reflected.

## 4. Discussion

This work is based on the idea of utilizing an abundant source of LCB and separating carbohydrates from lignin by applying two selective solvents that have been previously used to successfully fractionate other biomasses [24,45,46,47].

As a raw material, VS is a biomass with a relatively high content of lignin in comparison to other agricultural residues such as corn stover (11–14%) [48] or sugarcane bagasse (15–25%) [49]. This also means that the carbohydrate content of VS is not as high as in those agro-residues, which could be considered a disadvantage when the target of the process is sugars. However, the objective of this work is not only to recover the sugars but also to obtain a secondary stream rich in lignin, which could also be independently valorized. From this point of view, the overall composition of VS is well balanced and therefore, suitable for the proposed study.

Although the comparison of the results among various biomass types, different DES, and operating conditions may be challenging, the results found in the literature indicated that the DES mixtures chosen here could be effective when applied to VS since the published work reported significant rates of delignification (especially for ChCl:LA) and enhanced enzymatic digestibility using other agricultural residues. For example, using ChCl:LA at 1:2 in bagasse (at 130 °C for 2 h and 10% biomass concentration), Li et al. observed a solubility range of 8.6% to 47.9% for lignin [16]. Similarly, in the case of walnut and peach endocarp with ChCl:LA 1:2 (at 145 °C for 6 h at 200 rpm and 10% wt. biomass loading), the authors reported delignification values that ranged from 64.3% to 70.2% and sugar conversion rates that surpassed 90% [45]. Also, a maximum cellulose recovery of 82.7%, a xylan removal of 77.6%, a lignin removal of 61.9%, and a cellulose conversion yield of 73.4% were achieved after the pretreatment of corn stover with ChCl:LA 1:2 (at 130 °C for 2 h and 10% wt. biomass concentration) [46]. A similar range of delignification values (between 62.3% and 81.6%, depending on the variety of sugarcane crop) was obtained by Chourasia et al. [47] working with sugarcane bagasse and ChCl:LA 1:5. Moreover, Hossain and co-workers [24] observed that the pretreatment of rice straw with ChCl:EG improved the digestibility of cellulose from 21% to 87% and fractionated 74% by weight of lignin even if under severe temperature and time conditions. The results obtained with the DES pretreatment in this work are well aligned with the above values, confirming the adequacy of the selected solvents for the purposes of this study. Indeed, our results demonstrate the effectiveness of the ChCl-based DES pretreatment for the fractionation and valorization of vine shoot biomass. Thus, at the most favorable conditions, the DES pretreatment described here resulted in a substantial increase in sugar conversion yields compared to the untreated biomass, attaining 75.2% and 99.9% for cellulose and xylan, respectively, as well as a remarkable lignin removal of up to 56.2%.

The effectiveness of a pretreatment method largely hinges on choosing a technology that significantly influences biomass breakdown and sugar release [45]. In the present case, we chose two DESs with different features, which resulted in a different interaction with the biomass. DESs have the capacity to establish hydrogen bonds with cellulose, leading to its dissolution, apart from the internal hydrogen bonds within the DES structures. The presence of carbonyl oxygens within the DES-forming compounds allows them to participate in the formation of two hydrogen bonds, while hydroxyl oxygen can contribute to a hydrogen bond. Hence, LA molecules have the potential to establish four hydrogen bonds whereas EG molecules can form two. Additionally, ChCl can accept three H+ ions due to its amino group and the hydroxyl group within its structure can facilitate an additional hydrogen bond. Consequently, the hydroxyl groups of LA can establish two hydrogen bonds with the amino group of ChCl. Furthermore, ChCl has the capability to create one hydrogen bond with the hydroxyl group of cellulose, while lactic acid molecules have the potential to generate up to four hydrogen bonds with cellulose. This results in a total of five bonds formed with cellulose. In the context of EG, it can form two hydrogen bonds with ChCl and two with cellulose. It is noteworthy that due to the higher electronegativity of oxygen compared to nitrogen, the hydrogen bonds formed by the amine nitrogen atoms are comparatively weaker than those formed by the carbonyl oxygen [50,51]. Taking into consideration the limited number of hydrogen bonds with cellulose observed in the ChCl:EG system as opposed to ChCl:LA, it becomes apparent that cellulose dissolution is more pronounced in the DES formed with LA than in that involving EG, as it was experimentally confirmed in the present study. Furthermore, the hydrophilicity, polarity, acidity, and hydrogen-bonding ability of HBDs have been shown to be the most influential properties associated with their performance in biomass pretreatment in terms of delignification [46]. The reduction in the recalcitrant structure of lignocellulosic biomass, in particular the strong binding of carbohydrates to lignin, precedes lignin removal by DES. The degree of lignin removal depends on the degree of lignin/carbohydrate breakage and, to some extent, on the removal of hemicelluloses [22]. The results obtained here are consistent with previous studies which state that the solubility of cellulose in this type of DES is generally low, while the solubility of hemicellulose is high [52,53].

In the present work, the authors explored the impact of three variables on different parameters used to assess the effectiveness of the pretreatment. One of the studied variables, temperature, has been widely studied for similar pretreatments using other substrates, but the other two (particle size and solid loading) are usually not taken into account when designing the experimental plan. The outcomes of the present work emphasize the importance of these under-studied variables and indicate the next steps to investigate the application of DES biomass fractionation beyond the laboratory scale. Particularly, the use of a high solid loading of 30% in the DES pretreatment tests aims at evaluating the pretreatment performance under experimental conditions more realistic in terms of scaling up the process and more compatible with other pretreatment techniques that may involve solid contents above 10–15%. Additionally, the utilization of high solid-content pretreated materials in the subsequent step of EH would result in high-concentration sugar media for further fermentation/conversion, which is essential to increase the final product yield [53].

Concerning the particle size, it is known that a lower particle size may be beneficial to the enzymatic hydrolysis yield through an enlargement of the enzyme-accessible surface area [54]. However, as pointed out by the authors, it is necessary to consider overall balances, since submillimetre small particles may result in low carbohydrate recoveries, as occurred in the experiments reported herein (Figure 3), where the decrease in particle size from 2 to 0.5 mm caused reduced cellulose and xylan recoveries. Another factor advocating the use of larger particle sizes would be the reduction in the energy spent on size reduction operations. Our results so far show that smaller particle size is better in terms of enhanced sugar conversion and delignification extent, especially when combined with high solid loadings of 30%. Since the available surface area seems to be a crucial factor for an effective DES pretreatment, the use of technologies that combine mechanical and chemical effects, such as extrusion or ball milling [11,55], could be the right path to follow.

Regarding temperature, it was confirmed that it is a critical variable. For ChCl:EG, the pretreatment at 120 °C was not enough to cause significant alterations to the lignocellulosic fibers. As hinted in Section 3.4, the viscosity of ChCl:EG (35.7–48.6 mPas at 298.15 K) [39,56] may hinder heat and mass transfer during the reactions, deteriorating the pretreatment performance and resulting in very low values of carbohydrate conversion at the lower T tested of 120 °C. Thus, raising the temperature up to 150 °C can contribute to solving this problem and significantly improve the enzyme’s performance [33]. However, the same temperature of 150 °C was an excessive value for the pretreatment with ChCl:LA, which probably led to the formation of pseudo-lignin structures, which caused a noticeable decrease in the enzymatic accessibility. Pseudo-lignin, being rich in aromatic structure and more hydrophobic than natural lignin, has been reported to exert greater inhibition on enzymatic hydrolysis and impede the access of enzymes to cellulose active sites [27,57].

Furthermore, from all the variables analyzed, only temperature showed a noticeable effect on the thermogravimetric characteristics of the recovered solids enriched in lignin. Although the solid loading in the pretreatment and the type of washing agent were very determining for the delignification of VS, they did not influence the thermogravimetric properties of the recovered solids. This result would facilitate the intensification of the pretreatment by allowing the use of high solid loads and water washing without compromising the characteristics of the extracted lignin.

The TG/DTG profiles of the obtained lignin-rich solids showed a characteristic two-peak curve in the range of 200–400 °C, which was similar to the profiles obtained in other studies involving ChCl:LA and LCB [58,59]. Ji and co-workers [59] attributed the first peak to the decomposition of lignin with low molecular weight and their associated reactions, while the second peak corresponded to the cleavage of C-C bonds between lignin units. Following this hypothesis, the solids that show a higher peak between 150 and 300 °C would be composed of lower-weight lignin. In the present work, those are the solids recovered from the 150 °C DES pretreatment, which is in accordance with the greater effect exerted by the temperature on the DES biomass system already discussed.

The TGA analysis carried out in this work serves only as a preliminary assessment of the recovered lignin. They allowed us to determine the influence of the four variables studied (temperature, particle size, solid loading, and washing agent) on the extracted lignin. Nevertheless, additional exams using other analytical techniques would be necessary to fully characterize these solids and find the most suitable application for them according to their characteristics.

## 5. Conclusions

The physicochemical properties of the ChCl-based DESs have a significant influence on the process, enhancing its efficacy through an increased capacity to form hydrogen bonds with the primary components of the biomass. Among the investigated DES, ChCl:lactic acid 1:5 emerged as the most efficient solvent for vine shoot pretreatment. Carbohydrate conversion rates of up to 75.2% for cellulose and 99.9% for xylan were achieved. The incorporation of a suitable washing agent (a 50% acetone/water solution) further enhanced delignification with a significant increase of up to 31.5%. The analysis of the recovered lignin-enriched solids exhibits consistent thermogravimetric properties regardless of the applied pretreatment conditions. Further investigation is needed to adapt the process to high consistency while maintaining good efficiency.

In summary, this study presents an eco-friendly technology for efficient vine shoot biomass fractionation, yielding valuable fermentable sugars and facilitating lignin recovery. Insights into the ChCl-based deep eutectic solvent (DES) pretreatment mechanism are provided. However, considerations regarding DES recyclability, viscosity, and cellulase inhibition must be addressed for large-scale applications.

## Figures and Tables

**Table 1:** Values of all the conditions (temperature, solid loading, and size of the biomass to be treated) tested in the assays carried out with both evaluated DESs (ChCl:LA (1:5) and ChCl:EG (1:2)).

DES Solvent Pretreatment with ChCl:LA (1:5) and ChCl:EG (1:2)			
**Assay Conditions**	Temperature (°C)	Solid Loading (%)	Particle Size (mm)
120-5-2	120	5	2
120-5-0.5			0.5
120-10-2		10	2
120-10-0.5			0.5
120-30-2 *		30	2
120-30-0.5 *			0.5
150-5-2	150	5	2
150-5-0.5			0.5
150-10-2		10	2
150-10-0.5			0.5
150-30-2		30	2
150-30-0.5			0.5

* only tested with ChCl:LA (1:5).

**Table 2:** Composition of raw VS biomass in g/100 g of dry biomass, (%). Data represent mean values of triplicate analysis and standard deviation.

Component	Composition (g/100 g of Dry Biomass, (%))
Cellulose	32.3 ± 0.5
Hemicellulose	18.4 ± 0.3
*Xylan*	14.8 ± 0.3
*Galactan*	1.9 ± 0.06
*Arabinan*	1.0 ± 0.04
*Mannan*	0.7 ± 0.01
Acetyl groups	5.3 ± 0.05
Acid-insoluble lignin	26.0 ± 0.1
Acid-soluble lignin	1.6 ± 0.05
Ash	3.6 ± 0.3
Aqueous extractives	6.9 ± 0.1
Ethanol extractives	1.4 ± 0.2

**Table 3:** Solid recovery values (%) using Equation (1) obtained after the different pretreatment conditions using the DESs ChCl:LA (1:5) or ChCl:EG (1:2).

Assay Conditions	ChCl:LA (1:5)	ChCl:EG (1:2)
	Solid Recovery (%)	Solid Recovery (%)
120-5-2	69.2 ± 1.5	91.7 ± 0.2
120-5-0.5	60.2 ± 0.2	81.9 ± 0.0
120-10-2	65.0 ± 4.3	89.2 ± 0.0
120-10-0.5	66.0 ± 2.8	85.6 ± 5.1
120-30-2	66.6 ± 1.9	-
120-30-0.5	67.4 ± 3.0	-
150-5-2	73.6 ± 5.6	77.1 ± 2.2
150-5-0.5	69.4 ± 5.7	78.4 ± 0.2
150-10-2	62.9 ± 1.4	86.8 ± 0.0
150-10-0.5	65.6 ± 1.2	78.4 ± 0.0
150-30-2	58.4 ± 5.4	80.4 ± 1.7
150-30-0.5	70.4 ± 0.7	82.1 ± 0.4

**Table 4:** Effect of pretreatment conditions and washing agents (distilled water or 50% acetone/water) on sample composition (g/100 g dry biomass), component and solid recovery (%), and sugar conversion yield (cellulose and xylan yields (%)) after enzymatic hydrolysis of DES-pretreated vine shoots with (A) ChCl:LA (1:5) and (B) ChCl:EG (1:2).

Assay Conditions		ChCl:LA (1:5)		ChCl:EG (1:2)	
		120 °C-30%-0.5 mm		150 °C-30%-0.5 mm	
Washing agent		H_2_O	50% Acetone: H_2_O	H_2_O	50% Acetone: H_2_O
Composition analysis (g/100 g dry biomass)	Cellulose	40.2 ± 0.7	45.0 ± 1.8	36.0 ± 0.2	40.0 ± 0.6
	Xylan	8.5 ± 0.1	9.3 ± 0.3	13.8 ± 0.1	15.6 ± 0.0
	Acid-insoluble lignin	35.4 ± 0.5	29.6 ±1.0	35.9 ± 0.2	29.7 ± 1.4
Component recovery yield (%)	Cellulose	84.0 ± 3.8	82.0 ± 0.8	85.9 ± 0.4	84.3 ± 5.1
	Xylan	38.8 ± 1.8	42.0 ± 0.4	71.8 ± 0.3	71.9 ± 4.3
	Delignification	11.7 ± 3.9	31.5 ± 0.7	0.0 ± 0.5	23.5 ± 0.0
	Solid recovery	67.4 ± 3.0	71.5 ± 0.1	82.1 ± 0.4	88.5 ± 0.0
EH conversion yield (%)	Cellulose	56.4 ± 1.7	49.4 ± 2.1	54.6 ± 0.8	61.7 ± 3.2
	Xylan	77.6 ± 2.9	69.4 ± 4.2	60.3 ± 0.3	66.9 ± 2.5

**Table 5:** Mass loss (g/100 g dry biomass), Tmax (°C), and mass loss/min (g/100 g dry biomass/min) for the pretreated samples, raw vine shoot (VS) biomass, and a commercial lignin using ChCl:LA (1:5) and two washing agent (H_2_O and 50% acetone/water).

Assay		Mass Loss (g/100 g Dry Biomass)	Range T 260–280 °C		Range T 355–395 °C	
			Tmax (°C)	Mass Loss (%/min)	Tmax (°C)	Mass Loss (g/100 g Dry Biomass/min)
raw VS		80.8			357	13.9
Commercial lignin		61.8			395	7.5
ChCl:LA H_2_O	120-5-2	62.9	278	3.6	368	5.7
	120-5-0.5	64.6	271	4.8	371	4.8
	120-10-2	65.7	269	6.0	363	5.0
	120-10-0.5	63.9	273	4.2	369	5.6
	150-5-2	58.6	266	3.9	378	3.9
	150-5-0.5	56.7	273	2.4	376	4.4
	150-10-2	60.9	261	5.8	375	4.1
	150-10-0.5	57.7	269	3.0	375	4.2
ChCl:LA 50% acetone:H_2_O	120-30-0.5	64.6	274	3.8	365	5.2

## Data Availability

The original contributions presented in the study are included in the article; further inquiries can be directed to the corresponding author.

## References

[1] Hingsamer M., Jungmeier G., Lago C., Caldés N., Lechón Y. (2019). The Role of Bioenergy in the Emerging Bioeconomy. Biorefineries.

[2] Takkellapati S., Li T., Gonzalez M.A. (2018). An Overview of Biorefinery-Derived Platform Chemicals from a Cellulose and Hemicellulose Biorefinery. Clean Technol. Environ. Policy.

[3] Singh N., Singhania R.R., Nigam P.S., Dong C.D., Patel A.K., Puri M. (2022). Global Status of Lignocellulosic Biorefinery: Challenges and Perspectives. Bioresour. Technol..

[4] Gullón B., Eibes G., Dávila I., Vila C., Labidi J., Gullón P. (2017). Valorization of Vine Shoots Based on the Autohydrolysis Fractionation Optimized by a Kinetic Approach. Ind. Eng. Chem. Res..

[5] Senila L., Tenu I., Carlescu P., Corduneanu O.R., Dumitrachi E.P., Kovacs E., Scurtu D.A., Cadar O., Becze A., Senila M. (2020). Sustainable Biomass Pellets Production Using Vineyard Wastes. Agriculture.

[6] García-Galindo D., Dyjakon A., Villa-Ceballos F.C. (2019). Building Variable Productivity Ratios for Improving Large Scale Spatially Explicit Pruning Biomass Assessments. Energies.

[7] Yiin C.L., Yap K.L., Ku A.Z.E., Chin B.L.F., Lock S.S.M., Cheah K.W., Loy A.C.M., Chan Y.H. (2021). Recent Advances in Green Solvents for Lignocellulosic Biomass Pretreatment: Potential of Choline Chloride (ChCl) Based Solvents. Bioresour. Technol..

[8] Francisco M., Van Den Bruinhorst A., Kroon M.C. (2012). New Natural and Renewable Low Transition Temperature Mixtures (LTTMs): Screening as Solvents for Lignocellulosic Biomass Processing. Green Chem..

[9] Zhang J., Fu Y., Dong Y.Y., Wang D., Deng J., Shi Z., Yang J., Yang H. (2023). Pretreatment of Bamboo with Choline Chloride-Lactic Acid Integrated with Calcium Chloride Hydrates Deep Eutectic Solvent to Boost Bioconversion for Ethanol Production. Ind. Crops Prod..

[10] Shen X.J., Wen J.L., Mei Q.Q., Chen X., Sun D., Yuan T.Q., Sun R.C. (2019). Facile Fractionation of Lignocelluloses by Biomass-Derived Deep Eutectic Solvent (DES) Pretreatment for Cellulose Enzymatic Hydrolysis and Lignin Valorization. Green Chem..

[11] Sun X., Zhou Z., Tian D., Zhao J., Zhang J., Deng P., Zou H., Lu C. (2023). Acidic Deep Eutectic Solvent Assisted Mechanochemical Delignification of Lignocellulosic Biomass at Room Temperature. Int. J. Biol. Macromol..

[12] Liu Q., Yuan T., Fu Q.J., Bai Y.Y., Peng F., Yao C.L. (2019). Choline Chloride-Lactic Acid Deep Eutectic Solvent for Delignification and Nanocellulose Production of Moso Bamboo. Cellulose.

[13] Chen Y.L., Zhang X., You T.T., Xu F. (2019). Deep Eutectic Solvents (DESs) for Cellulose Dissolution: A Mini-Review. Cellulose.

[14] Yin X., Wei L., Pan X., Liu C., Jiang J., Wang K. (2021). The Pretreatment of Lignocelluloses With Green Solvent as Biorefinery Preprocess: A Minor Review. Front. Plant Sci..

[15] Loow Y.L., New E.K., Yang G.H., Ang L.Y., Foo L.Y.W., Wu T.Y. (2017). Potential Use of Deep Eutectic Solvents to Facilitate Lignocellulosic Biomass Utilization and Conversion. Cellulose.

[16] Li C., Huang C., Zhao Y., Zheng C., Su H., Zhang L., Luo W., Zhao H., Wang S., Huang L.J. (2021). Effect of Choline-Based Deep Eutectic Solvent Pretreatment on the Structure of Cellulose and Lignin in Bagasse. Processes.

[17] Oh Y., Park S., Jung D., Oh K.K., Lee S.H. (2020). Effect of Hydrogen Bond Donor on the Choline Chloride-Based Deep Eutectic Solvent-Mediated Extraction of Lignin from Pine Wood. Int. J. Biol. Macromol..

[18] Smink D., Kersten S.R.A., Schuur B. (2020). Recovery of Lignin from Deep Eutectic Solvents by Liquid-Liquid Extraction. Sep. Purif. Technol..

[19] Kalhor P., Ghandi K. (2019). Deep Eutectic Solvents for Pretreatment, Extraction, and Catalysis of Biomass and Food Waste. Molecules.

[20] Lynam J.G., Kumar N., Wong M.J. (2017). Deep Eutectic Solvents’ Ability to Solubilize Lignin, Cellulose, and Hemicellulose; Thermal Stability; and Density. Bioresour. Technol..

[21] Zhang C.W., Xia S.Q., Ma P.S. (2016). Facile Pretreatment of Lignocellulosic Biomass Using Deep Eutectic Solvents. Bioresour. Technol..

[22] Xu H., Kong Y., Peng J., Song X., Liu Y., Su Z., Li B., Gao C., Tian W. (2021). Comprehensive Analysis of Important Parameters of Choline Chloride-Based Deep Eutectic Solvent Pretreatment of Lignocellulosic Biomass. Bioresour. Technol..

[23] Satlewal A., Agrawal R., Bhagia S., Sangoro J., Ragauskas A.J. (2018). Natural Deep Eutectic Solvents for Lignocellulosic Biomass Pretreatment: Recent Developments, Challenges and Novel Opportunities. Biotechnol. Adv..

[24] Hossain M.A., Rahaman M.S., Yelle D., Shang H., Sun Z., Renneckar S., Dong J., Tulaphol S., Sathitsuksanoh N. (2021). Effects of Polyol-Based Deep Eutectic Solvents on the Efficiency of Rice Straw Enzymatic Hydrolysis. Ind. Crops Prod..

[25] Mamilla J.L.K., Novak U., Grilc M., Likozar B. (2019). Natural Deep Eutectic Solvents (DES) for Fractionation of Waste Lignocellulosic Biomass and Its Cascade Conversion to Value-Added Bio-Based Chemicals. Biomass Bioenergy.

[26] del Mar Contreras-Gámez M., Galán-Martín Á., Seixas N., da Costa Lopes A.M., Silvestre A., Castro E. (2023). Deep Eutectic Solvents for Improved Biomass Pretreatment: Current Status and Future Prospective towards Sustainable Processes. Bioresour. Technol..

[27] Chen Z., Jiang D., Zhang T., Lei T., Zhang H., Yang J., Shui X., Li F., Zhang Y., Zhang Q. (2022). Comparison of Three Ionic Liquids Pretreatment of *Arundo Donax* L. For Enhanced Photo-Fermentative Hydrogen Production. Bioresour. Technol..

[28] NREL (2012). Determination of Structural Carbohydrates and Lignin in Biomass: Laboratory Analytical Procedure (LAP) (Revised August 2012): Issue Date: 4/25/2008. https://www.nrel.gov/docs/gen/fy13/42618.pdf.

[29] Moreno A.D., Duque A., González A., Ballesteros I., Negro M.J. (2021). Valorization of Greenhouse Horticulture Waste from a Biorefinery Perspective. Foods.

[30] Zhai Q., Long F., Jiang X., Hse C.Y., Jiang J., Xu J. (2020). Facile and Rapid Fractionation of Bamboo Wood with a P-Toluenesulfonic Acid-Based Three-Constituent Deep Eutectic Solvent. Ind. Crops Prod..

[31] Chang L., Sun Y., Gan L. (2023). Insights into Cellulose Deconstruction and Pseudo-Lignin Formation during Deep Eutectic Solvent Treatment. Cellulose.

[32] Cui P., Ye Z., Chai M., Yuan J., Xiong Y., Yang H., Yao L. (2023). Effective Fractionation of Lignocellulose Components and Lignin Valorization by Combination of Deep Eutectic Solvent with Ethanol. Front. Bioeng. Biotechnol..

[33] Wang W., Lee D.J. (2021). Lignocellulosic Biomass Pretreatment by Deep Eutectic Solvents on Lignin Extraction and Saccharification Enhancement: A Review. Bioresour. Technol..

[34] Cardoza D., Contreras M.d.M., Lara-Serrano M., Morales-delaRosa S., Campos-Martín J.M., Romero I., Castro E. (2024). Sustainable Vine Shoots-to-Ethanol Valorisation by a Sequential Acid/Organosolv Pretreatment. Process Saf. Environ. Prot..

[35] Dávila I., Gullón P., Andrés M.A., Labidi J. (2017). Coproduction of Lignin and Glucose from Vine Shoots by Eco-Friendly Strategies: Toward the Development of an Integrated Biorefinery. Bioresour. Technol..

[36] Quintero L.P., de Souza N.P.Q., Milagres A.M.F. (2022). The Effect of Xylan Removal on the High-Solid Enzymatic Hydrolysis of Sugarcane Bagasse. BioEnergy Res..

[37] Paulsen Thoresen P., Lange H., Rova U., Christakopoulos P., Matsakas L. (2023). Role and Importance of Solvents for the Fractionation of Lignocellulosic Biomass. Bioresour. Technol..

[38] Li D., Qi L., Yang M., Gu Y., Xue Y., Chen J., He M., Yang G. (2023). Switchable Deep Eutectic Solvents for Lignin Dissolution and Regeneration. Polymers.

[39] Kroon. M.C., Casal. M.F., Van Den Bruinhorst A. (2013). Pretreatment of Lignocellulosc Biomass and Recovery of Substituents Using Natural Deep Eutectic Solvents/Compound Mixtures with Low Transtion Temperatures. Patent.

[40] Domínguez-Robles J., Tamminen T., Liitiä T., Peresin M.S., Rodríguez A., Jääskeläinen A.S. (2018). Aqueous Acetone Fractionation of Kraft, Organosolv and Soda Lignins. Int. J. Biol. Macromol..

[41] Moradi H., Farzi N. (2021). Experimental and Computational Assessment of the Physicochemical Properties of Choline Chloride/Ethylene Glycol Deep Eutectic Solvent in 1:2 and 1:3 Mole Fractions and 298.15–398.15 K. J. Mol. Liq..

[42] Jin W., Shen D., Liu Q., Xiao R. (2016). Evaluation of the Co-Pyrolysis of Lignin with Plastic Polymers by TG-FTIR and Py-GC/MS. Polym. Degrad. Stab..

[43] Xiang A., Ebdon J.R., Horrocks A.R., Kandola B.K. (2022). On the Utility of Thermogravimetric Analysis for Exploring the Kinetics of Thermal Degradation of Lignins. Bioresour. Technol. Reports.

[44] Muhammad N., Man Z., Bustam M.A., Mutalib M.I.A., Rafiq S. (2013). Investigations of Novel Nitrile-Based Ionic Liquids as Pre-Treatment Solvent for Extraction of Lignin from Bamboo Biomass. J. Ind. Eng. Chem..

[45] Li W., Amos K., Li M., Pu Y., DeBolt S., Ragauskas A.J., Shi J. (2018). Fractionation and Characterization of Lignin Streams from Unique High-Lignin Content Endocarp Feedstocks. Biotechnol. Biofuels.

[46] Liang X., Zhu Y., Qi B., Li S., Luo J., Wan Y. (2021). Structure-Property-Performance Relationships of Lactic Acid-Based Deep Eutectic Solvents with Different Hydrogen Bond Acceptors for Corn Stover Pretreatment. Bioresour. Technol..

[47] Chourasia V.R., Pandey A., Pant K.K., Henry R.J. (2021). Improving Enzymatic Digestibility of Sugarcane Bagasse from Different Varieties of Sugarcane Using Deep Eutectic Solvent Pretreatment. Bioresour. Technol..

[48] Alavijeh R.S., Shahvandi A., Okoro O.V., Denayer J.F.M., Karimi K. (2023). Biorefining of Corn Stover for Efficient Production of Bioethanol, Biodiesel, Biomethane, and Value-Added Byproducts. Energy Convers. Manag..

[49] Cai J., He Y., Yu X., Banks S.W., Yang Y., Zhang X., Yu Y., Liu R., Bridgwater A.V. (2017). Review of Physicochemical Properties and Analytical Characterization of Lignocellulosic Biomass. Renew. Sustain. Energy Rev..

[50] Zhang H., Lang J., Lan P., Yang H., Lu J., Wang Z. (2020). Study on the Dissolution Mechanism of Cellulose by ChCl-Based Deep Eutectic Solvents. Materials.

[51] Kwon G.J., Yang B.S., Park C.W., Bandi R., Lee E.A., Park J.S., Han S.Y., Kim N.H., Lee S.H. (2020). Treatment Effects of Choline Chloride-Based Deep Eutectic Solvent on the Chemical Composition of Red Pine (Pinus Densiflora). BioResources.

[52] Kumar A.K., Parikh B.S., Pravakar M. (2016). Natural Deep Eutectic Solvent Mediated Pretreatment of Rice Straw: Bioanalytical Characterization of Lignin Extract and Enzymatic Hydrolysis of Pretreated Biomass Residue. Environ. Sci. Pollut. Res..

[53] Gao W., Li Z., Liu T., Wang Y. (2021). Production of High-Concentration Fermentable Sugars from Lignocellulosic Biomass by Using High Solids Fed-Batch Enzymatic Hydrolysis. Biochem. Eng. J..

[54] Yang Y., Zhang M., Zhao J., Wang D. (2023). Effects of Particle Size on Biomass Pretreatment and Hydrolysis Performances in Bioethanol Conversion. Biomass Convers. Biorefinery.

[55] Yan M., Tian C., Wu T., Huang X., Zhong Y., Yang P., Zhang L., Ma J., Lu H., Zhou X. (2021). Insights into Structure and Properties of Cellulose Nanofibrils (CNFs) Prepared by Screw Extrusion and Deep Eutectic Solvent Permeation. Int. J. Biol. Macromol..

[56] Gajardo-Parra N.F., Cotroneo-Figueroa V.P., Aravena P., Vesovic V., Canales R.I. (2020). Viscosity of Choline Chloride-Based Deep Eutectic Solvents: Experiments and Modeling. J. Chem. Eng. Data.

[57] Schmatz A.A., Salazar-Bryam A.M., Contiero J., Sant’Anna C., Brienzo M. (2021). Pseudo-Lignin Content Decreased with Hemicellulose and Lignin Removal, Improving Cellulose Accessibility, and Enzymatic Digestibility. BioEnergy Res..

[58] Wang L., Li X., Jiang J., Zhang Y., Bi S., Wang H.-M. (2022). Revealing Structural and Functional Specificity of Lignin from Tobacco Stalk during Deep Eutectic Solvents Deconstruction Aiming to Targeted Valorization. Ind. Crops Prod..

[59] Ji Q., Yu X., Wu P., Yagoub A.E.-G.A., Chen L., Abdullateef Taiye M., Zhou C. (2021). Pretreatment of Sugarcane Bagasse with Deep Eutectic Solvents Affect the Structure and Morphology of Lignin. Ind. Crops Prod..

